# Does hand grip strength decrease in chronic obstructive pulmonary disease exacerbation? A cross-sectional study

**DOI:** 10.3906/sag-1811-22

**Published:** 2019-06-18

**Authors:** Zeynep TURAN, Özden ÖZYEMİŞÇİ TAŞKIRAN, Zeynep ERDEN, Nurdan KÖKTÜRK, Gülçin KAYMAK KARATAŞ

**Affiliations:** 1 Department of Physical Medicine and Rehabilitation, Faculty of Medicine, Koç University, İstanbul Turkey; 2 Department of Physical Medicine and Rehabilitation, Balıkesir Ayvalık State Hospital, Balıkesir Turkey; 3 Department of Pulmonary Medicine, Faculty of Medicine, Gazi University, Ankara Turkey; 4 Department of Physical Medicine and Rehabilitation, Faculty of Medicine, Gazi University, Ankara Turkey

**Keywords:** Chronic obstructive pulmonary disease, hand grip strength, 6-min walk distance, exacerbation, muscle strength

## Abstract

**Background/aim:**

Respiratory and peripheral muscle strength are reduced in chronic obstructive pulmonary disease (COPD). There is a well-known correlation between handgrip strength (HGS) and strenght extremity muscles. Our aim in this study was to measure HGS and investigate the related factors in COPD patients with exacerbation.

**Materials and methods:**

Subjects with COPD exacerbation (n = 101) and stable COPD (n = 22), and subjects without COPD (n = 201), were enrolled in this study. Age, sex, and body mass index were similar. HGS was measured using a Vigorimeter. Pulmonary function tests and 6-min walk tests were performed.

**Results:**

The mean HGS was significantly lower in subjects with COPD exacerbation than those with stable COPD and subjects without COPD. The mean HGS was similar between stable COPD and non-COPD subjects. The mean 6-min walk distance (6MWD) was significantly lower in subjects with COPD exacerbation than stable COPD. There was a significant correlation between HGS and 6MWD but no correlation between HGS and pulmonary function tests.

**Conclusion:**

In subjects with COPD exacerbation, the HGS was lower than that of stable COPD patients, and this difference was not explained by age, comorbidities, severity of obstruction, or smoking. Physical inactivity and steroid use during exacerbation might be possible factors affecting HGS. HGS was moderately correlated with 6MWD in cases of exacerbation. It may be used as a measure of muscle performance in COPD exacerbation, especially when the 6-min walk test cannot be performed.

## 1. Introduction

Chronic obstructive pulmonary disease (COPD) has several systemic manifestations, including skeletal muscle dysfunction, which increases the risk of morbidity and mortality (1). The strength of skeletal and respiratory muscles is reduced in COPD compared to the normal population (1–4). The weakness of the quadriceps muscle is observed in 70% of subjects with chronic lung disease (3) and is more prominent than weakness of upper extremity muscles such as the pectoralis major and latissimus dorsi in COPD (5). Maximal strength of the diaphragm is 30% to 40% lower in cases of COPD (1). 

Handgrip strength (HGS), which is a simple and inexpensive measurement, is not only a direct measure of hand muscle strength but also a good indicator of overall muscle strength in elderly subjects (6,7). It was shown to be correlated with the strength of other muscles such as the quadriceps (8), upper limb muscles (9), and respiratory muscles (8,9) in COPD. Low HGS was independently associated with a higher risk for exacerbations in smokers with stable COPD (10). It was demonstrated that HGS was associated with cardiac function (11) and walking distance (8) in COPD cases.

The 6-min walk test (6MWT) is an objective and inexpensive method used to assess submaximal exercise capacity. Muscle strength in the lower limbs was shown to be an important factor in determining the 6-min walk distance (6MWD) (8,12). The 6MWD was also significantly correlated with handgrip force in individuals with stable COPD (8,12,13).

To our knowledge, there is no study comparing the handgrip strength of COPD subjects during exacerbation with those of stable COPD patients and subjects without COPD. The aim of this study was to measure HGS and investigate the associated factors, including 6MWD, in subjects with acute exacerbation of COPD.

## 2. Materials and methods

This cross-sectional study was approved by the Gazi University Ethics Committee (Date/No: 09.03.2015/123). All participants gave their written informed consent for the study.

### 2.1. Participants

Participants were recruited among subjects with COPD registered in pulmonary rehabilitation medical records between January 2010 and December 2014. Subjects with acute exacerbation of COPD (n = 101) and subjects with stable COPD (n = 22) were enrolled in the study. The diagnosis of COPD was made according to the Global Initiative for Chronic Obstructive Lung Disease (GOLD) criteria (14). For the non-COPD group, individuals without COPD (n = 201) were recruited among patients admitted to the physical medicine and rehabilitation outpatient clinic for other problems such as meniscal degeneration, knee osteoarthritis, and low back pain. 

The exclusion criteria were the presence of cancer, neurologic or rheumatologic disease, advanced heart disease (New York Heart Association Classification stages III or IV), or any other condition that might adversely influence upper extremity functions and the cooperation of the individual. 

### 2.2. Measures

Demographic variables including age, sex, height, weight, body mass index (BMI), years of education, and systemic illnesses were recorded for all subjects. The duration of COPD, smoking status, pulmonary function, other related clinical parameters, 6MWD, and length of inpatient stay (for the exacerbation group) were recorded for the subjects with COPD.

Handgrip strength was measured using the handheld Vigorimeter apparatus (Riester, Dynatest, Jungingen, Germany) according to the procedure recommended by the American Society of Hand Therapists (15). After the explanation of the procedure, subjects were seated placing their arms by their sides with the elbow flexed to 90°, the forearm mid-prone, and the wrist in neutral position. Subjects were asked to squeeze the Vigorimeter with maximal effort to measure maximal voluntary HGS (in bars) for both the dominant and the nondominant hand. Standard verbal encouragement was given to every patient. Three trials were performed with a 1-min rest between trials and the highest value was used for the analysis. 

Pulmonary function tests were performed using a Sensormedics Vmax Series 20C Respiratory Analyzer according to the criteria of the European Respiratory Society for COPD subjects (16). Forced expiratory volume in 1 s (FEV1), forced vital capacity (FVC), and FEV1/FVC ratios were recorded. 

The 6MWT was performed with COPD subjects according to the American Thoracic Society guidelines (17) in a 30-m corridor. The subjects were asked to walk as much distance as they could within 6 min. The 6MWD was recorded in meters. 

Statistical analysis was performed using SPSS 20.0 for Windows (IBM Corp., Armonk, NY, USA). Continuous variables were presented as means and standard deviations. Categorical data were presented as percentages. Statistical analysis was performed using ANOVA with post hoc Tukey analysis and Student’s t-test as appropriate. Pearson’s correlation coefficient was computed to analyze the correlation between HGS and other parameters in COPD subjects with exacerbation. The comparison of categorical data was performed using Fisher’s exact test. The variables of the COPD subjects with exacerbation who could and could not complete the 6MWT were evaluated using Student’s t test or the chi-square test for continuous and categorical variables, respectively. Missing data were removed and the remaining data were analyzed. The sample size was calculated using G*Power with regard to Vilaro et al. (18) and it was seen that the recruitment of 119 stable and 119 exacerbated subjects was necessary. Statistical significance was accepted as P < 0.05.

## 3. Results

One hundred and eleven subjects with COPD exacerbation, 27 subjects with stable COPD, and 252 non-COPD subjects were potentially eligible for the study. After the confirmation of the eligibility of the participants, 101 subjects with COPD exacerbation, 22 subjects with stable COPD, and 201 non-COPD subjects were enrolled. The Figure shows the flow diagram.

**Figure 1:**
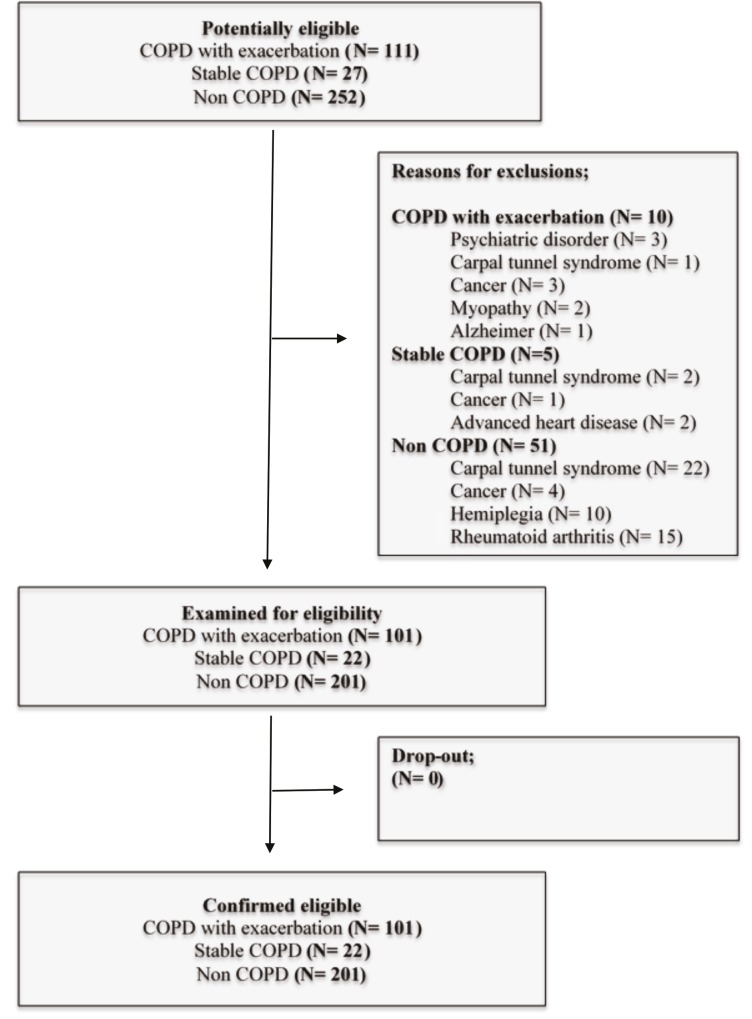
Flow diagram of the study.

The demographic and clinical features of the subjects are presented in Table 1. Age, sex, and BMI were similar among the groups. Education years were higher in the stable COPD subjects than the other groups (P = 0.004). The prevalence of hypertension and heart disease was significantly different among the groups and the difference was due to the non-COPD group. Subgroup analysis did not reveal any statistically significant differences between the subjects with COPD exacerbation and stable COPD.

**Table 1:** Demographic characteristics of the subjects.

	COPD – exacerbated (n=101),mean ± SD or %	COPD – stable (n = 22),mean ± SD or %	Non-COPD (n = 201),mean ± SD or %	P
Age (years)	68.3 ± 9.1	65.6 ± 8.8	66.6 ± 6.4	0.082
Men (%)	74	82	82	0.417
BMI (kg/m2)	26.36 ± 7.67	27.78 ± 5.86	27.90 ± 3.31	0.052
Education (years)	6.9 ± 4.6	8.8 ± 5.2*	6.1 ± 2.9	0.004
Heart disease (%)	46	36	10*	<0.001
Hypertension (%)	56	46	23*	<0.001
Diabetes mellitus (%)	20	14	25	0.314

COPD: Chronic obstructive pulmonary disease. SD: Standard deviation. BMI: Body mass index. *: The group from which the difference originates.

Table 2 demonstrates clinical features of the COPD subjects. The respiratory parameters (FEV1, FEV1/FVC) of the exacerbation group were lower than those of the group with stable COPD; however, the difference did not reach statistical significance. 

**Table 2:** Clinical features of COPD subjects.

	COPD – exacerbated (n = 101), mean ± SD or %	COPD – stable (n = 22), mean ± SD or %	P
COPD duration (years)	7.6 ± 6.7	8.4 ± 6.9	0.661
FEV1 (%)	38.9 ± 14.6(n = 90)	45.8 ± 18.6(n = 19)	0.108
FEV1/FVC	53.9 ± 10.3(n = 90)	58.9 ± 12.5(n = 19)	0.084
Stages (GOLD), %IIIIIIIV	(n = 90)1.118.947.832.2	(n = 19)5.221.157.915.7	0.258
Smoking status (%)	26	24	0.936
Smoking (pack years)	54.7 ± 35.2	57.3 ± 31.0	0.779

COPD: Chronic obstructive pulmonary disease. SD: Standard deviation. FEV1: Forced expiratory volume in 1 s. FVC: Forced vital capacity.

HGS measurements of the dominant and nondominant hands and 6MWD parameters are given in Table 3. The mean HGS was significantly lower in the COPD subjects with exacerbation than the stable COPD and non-COPD subjects (P < 0.001). The mean 6MWD was significantly lower in subjects with COPD exacerbation than those with stable COPD (P = 0.003). 

**Table 3:** Measurements of handgrip strength and 6MWD.

	COPD – exacerbated (n = 101),mean ± SD or %	COPD – stable (n = 22),mean ± SD or %	Non-COPD (n = 201),mean ± SD or %	P
HGS – dominant (bar)	0.47 ± 0.17*	0.57 ± 0.16	0.55 ± 0.16	<0.001
HGS – nondominant (bar)	0.44 ± 0.16*	0.55 ± 0.16	0.52 ± 0.16	<0.001
6MWD (m)	232.4 ± 149.3	335.9 ± 120.0	NA	0.003

COPD: Chronic obstructive pulmonary disease. SD: Standard deviation. HGS: Handgrip strength. 6MWD: Six-minute walk distance. NA: Not applied *: The group from which the difference originates.

Table 4 shows the correlations between HGS (the mean of dominant and nondominant HGS) and the other parameters in COPD subjects with exacerbation. HGS demonstrated a moderate correlation with 6MWD and a weak negative correlation with age and length of inpatient stay. There was no correlation between HGS and FEV1 or COPD duration. 

**Table 4:** Correlation between mean handgrip strength and other parameters in COPD subjects with exacerbation.

		6MWD	Age	COPD duration	BMI	FEV1	LOS
HGS (bar)	r*	0.516	–0.250	–0.158	0.086	-0.070	–0.247	P**	<0.001	0.012	0.134	0.395	0.509	0.015
	6MWD (m)	r		–0.269	–0.249	0.043	0.158	–0.151	P		0.007	0.017	0.670	0.137	0.141
Age (years)		r			0.188	–0.151	0.252	–0.107	P			0.076	0.136	0.017	0.302
	COPD duration (years)	r					-0.114	0.233	P					0.311	0.031
BMI (kg/m2)		r					0.273	–0.083	P					0.010	0.422
	FEV1 (%)	r						–0.088	P						0.422

HGS: Handgrip strength. 6MWD: Six-minute walk distance. COPD: Chronic obstructive pulmonary disease. BMI: Body mass index. FEV1: Forced expiratory volume in 1 s. LOS: Length of inpatient stay *r: Pearson correlation. **: P < 0.05 is significant.

The subjects with COPD exacerbation were divided according to their ability to complete the 6MWT. Twenty subjects could not even walk and 23 subjects stopped walking before 3 min, while 58 subjects were able to complete the test. Table 5 shows the characteristics of two groups (completed and not completed). The subjects who could not complete the 6MWT were older, and women with longer length of stay and COPD duration had lower HGS. BMI and respiratory parameters were not different between the two groups.

**Table 5:** Comparison of COPD subjects with exacerbation who could and could not complete the 6MWT.

	Completed (n = 58), mean ± SD or %	Not completed (n = 43), mean ± SD or %	P
Age (years)	67 ± 8	71 ± 10	0.033
Men (%)	68	32	<0.001
BMI (kg/m2)	27.3 ± 6.8	25.1 ± 8.7	0.156
COPD duration (years)	6 ± 6	9 ± 7	0.043
FEV1 (%)	40.6 ± 13.1	36.5 ± 16.3	0.193
Length of inpatient stay (days)	12 ± 6	15 ± 7	0.033
HGS (bar)	0.53 ± 0.14	0.36 ± 0.13	<0.001
6MWD (m)	317 ± 99	31 ± 48	<0.001

SD: Standard deviation. BMI: Body mass index. COPD: Chronic obstructive pulmonary disease. FEV1: Forced expiratory volume in 1 s. HGS: Handgrip strength. 6MWD: Six-minute walk distance.

## 4. Discussion

In this study, COPD subjects with exacerbation showed significantly lower HGS compared to the stable COPD and non-COPD subjects. In the COPD subjects with exacerbation, the mean HGS showed a moderate correlation with 6MWD and a weak negative correlation with age and length of stay. Nearly half (43%) of the COPD subjects with exacerbation could not complete the 6MWT and had lower HGS than the individuals who completed the test.

HGS was measured in cases of stable COPD in previous studies; Shah et al. found significantly lower HGS and endurance (9), whereas Heijdra et al. found similar HGS in patients with stable COPD and age-matched healthy subjects (19). Similar to Heijdra et al., our results revealed comparable HGS in stable COPD and non-COPD subjects. In a cross-sectional study, Martinez et al. found that a 1-kg reduction in HGS was associated with an increased risk of exacerbation by 5% in stable COPD (10). They did not measure HGS directly in the exacerbation period. Vilaro et al. observed that HGS was weaker in COPD patients with exacerbation than those with stable COPD. In our study, we primarily enrolled COPD subjects with exacerbation and found that their muscle strength was lower than that of stable COPD and non-COPD subjects. 

Several factors related to skeletal muscle dysfunction in COPD have been described, including comorbidities, smoking, aging, inactivity, exacerbation, hypoxia, systemic oxidative stress, systemic inflammation, and medications such as corticosteroids (20–22). In our study, age was similar among the three groups; the mean age of the subjects with COPD exacerbation was not different from that of the stable COPD or non-COPD subjects. Therefore, lower HGS in the exacerbation group compared to the subjects with stable COPD could not be attributed to older age. We observed that presence of chronic diseases, smoking status, and pulmonary function tests were similar in the subjects with exacerbated and stable COPD. The similarity of these factors in our study suggested that we exclude them as confounding parameters for lower HGS in exacerbation.

Systemic corticosteroids used in exacerbated or advanced COPD can induce both acute and chronic myopathy (20–23). In our study, all the patients with exacerbation used oral steroids for a short period, beginning with 32 mg decreasing slowly and ceasing at 10 days, which was the standardized treatment protocol. The steroids used during the treatment of exacerbation might have contributed to the reduced HGS in our exacerbation group, as well.

Physical inactivity due to breathlessness and old age (20) contributes to muscle atrophy and weakness in COPD subjects (1,22). In our study, the subjects with COPD exacerbation demonstrated lower levels of physical activity (shorter 6MWD) than the similarly aged stable COPD subjects. The subjects who could not complete the 6MWT in exacerbation were older and had weaker muscle strength, a longer duration of disease, and a longer hospital stay, all of which indicated higher disease severity. This reduction in physical activity in COPD is in accordance with the literature. Physical inactivity further decreases muscle strength and hence increases disability (24). Reduction in muscle strength is not limited to lower limb muscles; it involves all muscles (1,22). The correlation found between physical activity and HGS in our study is also supported by a direct relationship reported between HGS and physical activity in previous studies of COPD (25) and community-dwelling individuals (24,26). HGS has been shown to be a strong and independent predictor of exercise capacity in patients with heart failure (11). This supports our finding that 6MWD, a measure of functional capacity, is significantly correlated with HGS in COPD.

Skeletal muscle dysfunction seen in COPD is not homogeneous among different muscles (1). The strength of lower extremity muscles is more severely affected than upper extremity muscles and handgrip (2,8). This discrepancy is explained by the frequent use of the upper extremities in daily activities; thus, upper extremity muscle strength is relatively conserved (8). In light of these observations, it can be predicted that slight changes in HGS might be associated with larger changes in lower extremity muscle strength. It is suggested that grip strength measurements may help to identify patients at risk of deterioration of health (6,20). In our study, reduced HGS was correlated with a longer hospital stay and shorter 6MWD, which are indicators of reduced health status. 

As a predictor of disease severity, pulmonary function tests revealed lower FEV1 and FEV1/FVC during exacerbation than stable COPD in our study; however, this difference did not reach statistical significance. This may be due to the small sample size of stable COPD patients in our study. Cortopassi et al. and Shah et al. found a significant association between HGS and pulmonary function tests including FVC and FEV1 in stable COPD subjects (9,11). However, similar to our study, Kaymaz et al. (25) and Sirguroh and Ahmed (27) did not find any significant association between HGS and the severity of airway obstruction. Bernard et al. found that the relationship of airflow obstruction (FEV1) is weaker with quadriceps strength than shoulder girdle muscles (5). It is well known that the severity of obstruction (O) alone is not a good indicator of disease severity, but BMI (B), severity of dyspnea (D), and 6MWD (exercise capacity - E) are other important parameters and hence the BODE index is widely used. In our study population, despite a similar BMI and severity of obstruction, the 6MWD was shorter in COPD patients with exacerbation. 

The 6MWD is reduced by several diseases including COPD (13) and is significantly correlated with HGS (8). For a 10-kg increment in HGS, subjects walk approximately 14 m farther (13). The 6MWT can be performed with subjects who cannot use a cycle ergometer or treadmill (13). However, some people cannot perform the 6MWT, either. In this study, 20 subjects could not even walk and 23 subjects stopped before 3 min; these subjects were older, had a longer duration of COPD, and had a longer hospital stay. For subjects who cannot perform the 6MWT, HGS might provide information about overall muscle endurance and capacity. 

The small sample size is a limitation of our study. The results of our study cannot be generalized to all COPD subjects, especially those in the stable period.

Our study showed that grip strength was lower in subjects with COPD exacerbation compared to stable COPD and non-COPD subjects. The difference observed in HGS between exacerbated and stable COPD was not explained by age, comorbidities, the severity of obstruction, or smoking. Physical inactivity and steroid use during exacerbation might be the factors affecting low HGS in exacerbation. 

The moderate correlation between HGS and 6MWD during exacerbation led us to suggest that HGS might provide some information about overall muscle strength and functional capacity in subjects with COPD exacerbation. This might be clinically helpful, especially for subjects who cannot walk or complete the 6MWT.
